# Central nervous system Cryptococcoma mimicking demyelinating disease: a case report

**DOI:** 10.1186/s12883-020-01880-4

**Published:** 2020-08-12

**Authors:** Jie Wei, Xiang-Yu Li, Yue Zhang

**Affiliations:** 1 Department of neurology, 905th hospital of PLA Navy, No 1328 Huashan Road, Changning District, Shanghai, 200052 China; 2 Department of Laboratory Medicine, Huashan Hospital North, Fudan University, No 108 Luxiang Road, Baoshan District, Shanghai, 201907 China; 3 Department of Neurology, Huashan Hospital, Fudan University, No.12 Wulumuqi Road, Jing’an District, Shanghai, 200040 China

**Keywords:** Central nervous system cryptococcosis, Cryptococcomas, Tumefactive demyelinating lesion, Corticosteroid

## Abstract

**Background:**

Cerebral cryptococcomas is a rare form of central nervous system cryptococcosis. Most previous cases were mistaken for neoplasm before surgery. We present a case of cerebral cryptococcomas whose radiological profiles resembled demyelinating disease, especially tumefactive demyelinating lesion.

**Case presentation:**

A 40-year-old male was admitted for 1-week-long unconsciousness. Brain MRI revealed a rim-enhanced mass within the corpus callosum body. Central nervous system demyelinating disease was suspected. Empirical corticosteroid treatment led to some improvement, but his condition deteriorated 2 months later. Brain MRI revealed punctate new foci. *Cryptococcus neoformans* was detected in cerebrospinal fluid. Cryptococcus antigen test was positive in his current and previous cerebrospinal fluid samples. The patient died despite standard antifungal treatment.

**Conclusion:**

Diagnosis of cerebral cryptococcomas is challenging. It may mimic demyelinating diseases.

## Background

Central nervous system (CNS) cryptococcosis results from infection with the yeast fungus cryptococcus. A large proportion of patients with CNS cryptococcosis are immunosuppressed individuals, such as those with AIDS. Meningitis or meningoencephalitis is the most common form of the disease. Parenchymal involvements, including cryptococcomas and gelatinous pseudocysts have been occasionally reported [1–3]. Cryptococcoma is parenchyma granuloma caused by cryptococcal organisms. It is more common in immunocompetent host than in immunosuppressed individuals. *Cryptococcus gattii* is a more common causative pathogen than *Cryptococcus neoformans* [4]. Diagnosis of cryptococcoma is challenging, especially when it appears as a solitary lesion. In previous literature, most cryptococcomas were mistaken for neoplasms before surgery [2]. .As some special demyelinating diseases may look similar to neoplasms, we assume cryptococcoma should also be considered a differential to demyelinating diseases. Tumefactive demyelinating lesion (TDL) is a locally aggressive form of demyelination, usually manifesting as a solitary lesion. It tends to be large and ring-enhanced on imaging. Corticosteroid is effective in this condition. Here we report a case of CNS cryptococcoma whose radiological profiles and early response to corticosteroid resembled demyelinating disease. Initial positive response to corticosteroid was followed by deterioration and death.

## Case presentation

A previously healthy 40-year-old male was admitted to a hospital in Yinshang An’hui province on April 24, 2016. About 1 week before admission, the patient was found to be apathetic, uncommunicative and slow to move at home. Soon after that, he became unresponsive and bedridden and thus was sent to the hospital where feeding tube and urinary catheter were placed. Brain computed tomography (CT) revealed a hypodense lesion in the corpus callosum. Patient’s consciousness level continued to decline during admission. When he was referred to 905th hospital, Navy, PLA, he was in vegetative state. Both axial muscles and appendicular muscles were rigid; the arms were in flexion position and legs were in extension position.

**Fig. 1:**
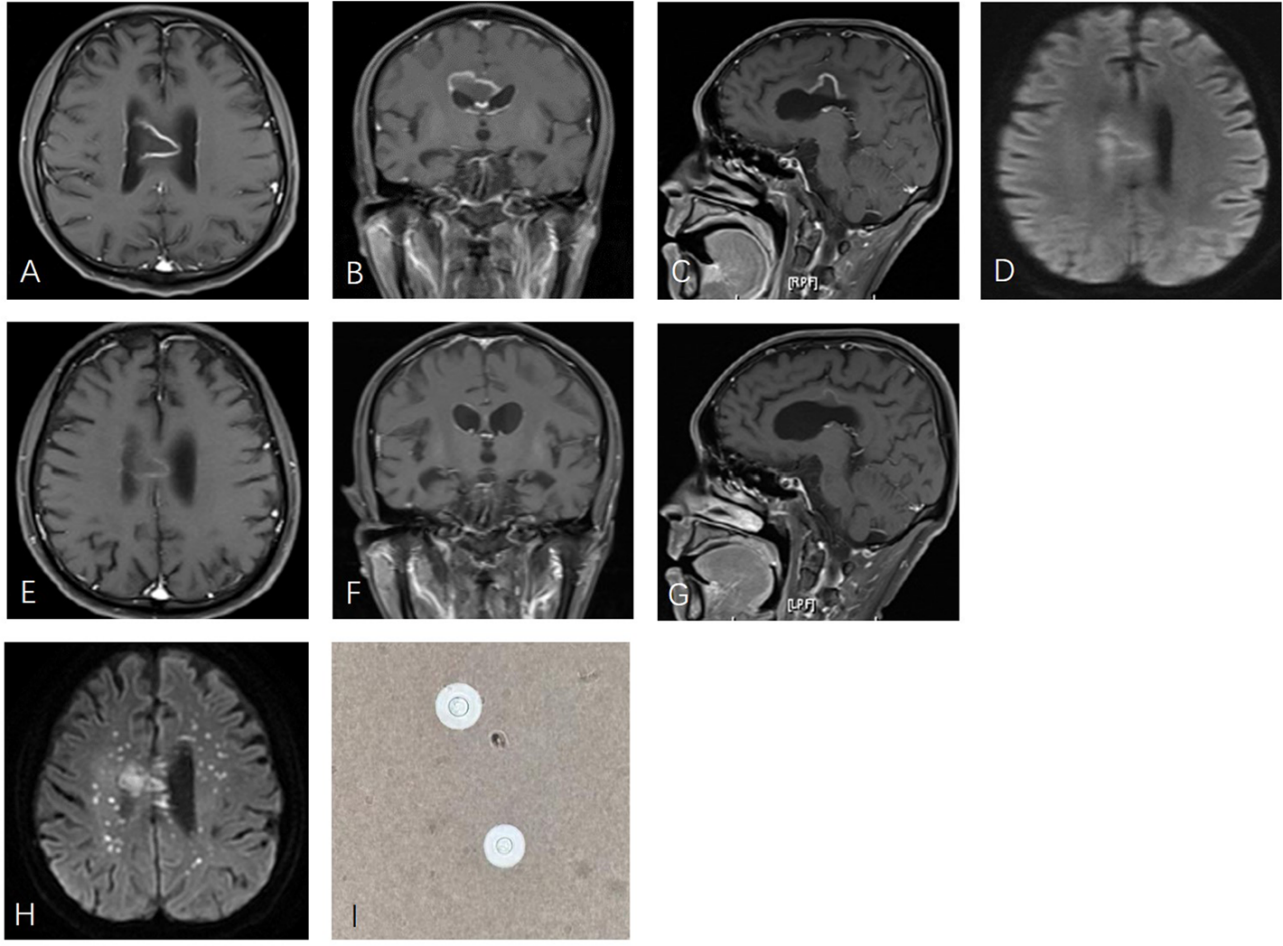
Axial postcontrast T1-weighted MR (**a**-**c**), demonstrated a rim-enhanced corpus callosum mass surrounded by vasogenic edema. Initial diffusion weighted images (**d**) showed rim hyperintensity but no restricted diffusion within the mass. Forty-two days after corticosteroid treatment, the mass shrank and the enhancement decreased (**e**-**g**). Seventy days after treatment, diffusion weighted images (**h**) revealed multiple punctate foci in addition to the corpus callosum mass. *Cryptococcus neoformans* was found in CSF with India ink stain (**i**).

Body temperature was 36.5 °C. Heart rate was 80 beats per minute. Blood pressure was 135/78 mmHg. Glasgow coma scale was scored 7 (eye opening: 3, verbal response: 1; motor response: 3). Pupil size was 3.5 mm in diameter bilaterally. Pupillary reaction to light was brisk. Corneal reflex was present. Axial and appendicular muscle tone increased. Upper extremities flexed. Lower extremities extended. Babinski and Chaddock signs were positive bilaterally. Blood leukocyte count was 3.79*10^9/L. N% was74.34%. C-reactive protein was 6.07 mg/L (normal range: 0–8.2). Liver and renal function were normal. Erythrocyte sediment rate was 21 mm/h. Serological testing was negative for HIV, syphilis, hepatitis B and C. anti-nuclear antibody, anti-cardiolipin antibody, anti-neutrophil cytoplasmic antibodies and T-SPOT test were negative. Lumbar puncture revealed opening pressure of 50mmH_2_O. Cerebrospinal fluid (CSF) was transparent and colorless. CSF protein elevated to 3.64 g/L (normal range: 0.15–0.45 g/L). CSF glucose level was 2.6 mmol/L (normal range: 2.4–4.5,) and simultaneous blood glucose was 5.38 mmol/L. CSF chloride level was 123 mmol/L (normal range: 120–132). Leukocyte count was 0 × 106/L. Oligoclonal band, aquaporin 4, myelin oligodendrocyte glycoprotein and glial fibrillary acidic protein antibodies were negative. Bacterial culture and Indian ink stain were negative. CSF sediment was cultured on Sabouraud’s agar for 72 h and no fungi was found. Histopathological analysis of the CSF with Wright staining did not find malignant cells. Lung CT revealed pulmonary nodules in the lower lobes. Brain MRI revealed a homogenous solid mass within the corpus callosum body. It was hypointense on T1W1 and isointense on T2 FLAIR. Post-contrast T1WI showed rim-enhancement and vasogenic edema (Fig. 1a-c).

Neoplasm was considered, but acute onset and rapid disease progression were not consistent with neoplastic infection. TDL is a special demyelinating disease whose characteristic MR finding is a solitary tumor-like lesion greater than 2 cm with ring-enhancement [5]. Brain abscess was not considered because diffusion weighted imagine (DWI) did not show restricted diffusion (Fig. 1d). On the assumption that corticosteroid may ameliorate demyelinating disease, but may aggravate infectious disease, while having little impact on neoplasms, empirical corticosteroid treatment (methylprednisolone 500 mg once daily) was initiated on April 26. Brain biopsy would be the option if improvement could not be achieved or symptoms continued to deteriorate.

Three days later, the patient showed response to verbal stimuli. One week later (on May 4), he was alert, oriented and cooperative. Speech was slurred. Muscle tone was stiff in the left extremities but was normal in the other parts of the body. Muscle strength was measured as 3/5 in the left leg and 4/5 in other extremities. Babinski sign was positive in the left. Feeding tube and urinary catheter were removed. On May 10, 2 weeks after corticotherapy, he was able to speak fluently and move with aid. Blood glucose was 6.6 mmol/l. Liver function, renal function and electrode were normal. When he was discharged on May 19, he could speak and walk normally. No obvious neurologic deficits remained. Oral prednisone of 60 mg daily was prescribed and was tapered by 5 mg every week. A repeat MRI on June 8 demonstrated lesion shrinkage and reduced rim enhancement (Fig. 1e-g). His condition remained stable until July 2016 when he became lethargic and apathetic again. MRI revealed punctate new foci in the centrum semiovale in addition to the corpus callosum mass (Fig. 1h). Lumbar puncture revealed open pressure of 250mmH_2_O. CSF glucose decreased to 2.03 mmol/l with simultaneous blood glucose of 9.11 mmol/l. CSF protein was elevated to 0.86 g/L, which was lower than that in the first lumbar puncture. Chloride was 118 mmol/L. Leucocyte count was 1*10^6/L. *Cryptococcus neoformans* was detected in the CSF by Indian ink staining. (Fig. 1i) CSF cryptococcus antigen test was positive at 1:640. Previously collected and stored CSF was tested for cryptococcus antigen and found to be positive as well. Intravenous amphotericin B 25 mg/d was given initially and then gradually increased to 50 mg/d. Oral flucytosine 6 g/d was also administered. However, his consciousness level declined rapidly. One week after treatment, he became comatose. Two weeks later, the patient developed status epilepticus. His relatives withdrew treatment based on his condition and the patient died.

## Discussion and conclusions

Cryptococcosis is an infectious disease caused by invasive encapsulated yeasts in the genus cryptococcus, including *Cryptococcus neoformans* and *Cryptococcus. gattii* [6]. Cryptococcosis may manifest as meningitis, meningoencephalitis, hydrocephalus, dilation of the perivascular spaces, cryptococcomas and gelatinous pseudocysts [1]. Meningitis and meningoencephalitis are the most common manifestations usually caused by *Cryptococcus neoformans* in immunosuppressed patients. Cryptococcomas are granulomas caused by cryptococcus within the brain parenchyma. They are more common in immunocompetent patients and the causative agent is usually *Cryptococcus gattii* [7]. In this case, no evidence of HIV infection was identified, but the pathogen was *Cryptococcus neoformans.*

The diagnosis of CNS cryptococcoma is challenging. Patients with cryptococcoma can be either immunosuppressed or immunocompetent. CSF profiles can be normal or show unspecific changes. Lung CT scans do not always show typical pulmonary changes [6, 8]. On brain MRI, the lesion may present as low signals on T1 weighted images and high signals on T2 weighted images or T2 FLAIR. Post contrast T1 weighted images can be variable, ranging from no enhancement to peripheral nodular enhancement [9]. But these findings are not specific [10]: Glioma and tumefactive demyelination may have similar radiological manifestation. Most previous cases, whether immunosuppressed or immunocompetent, were misdiagnosed as neoplasms before brain biopsy or resection [8, 10]. In this case, fever was not recorded and regular laboratory tests of blood and CSF did not show signs of infection during the first admission: That is why we ignored the possibility of infectious disease at first. The unexpected response to corticosteroid further interfered with diagnosis. Similar case reports were rare. Hagan JE, et al. reported a patient with cryptococcoma received dexamethasone before surgery because the lesion was mistaken for glioma initially. Corticotherapy led to some improvement, but after the diagnosis was confirmed dexamethasone was soon suspended [11]. We assume temporary clinical and radiological alleviation may result from anti-inflammatory and anti-edematous effect of corticosteroids. But corticosteroids should not be used in cryptococcosis, even as adjuvant treatment [12].

In conclusion, we describe an immunocompetent patient with CNS cryptococcoma misdiagnosed as demyelinating disease. Corticosteroid alleviated symptoms temporarily but led to death. Diagnosis of CNS cryptococcoma is difficult, and the radiological profile or short-term response to corticosteroid can be deceptive. With regard to rim-enhancing mass, cryptococcoma should be included in differential diagnosis list.

## Data Availability

Not applicable.

## Abbreviations

CNS: Central nervous system
CSF: Cerebrospinal fluid
TDL: Tumefactive demyelinating lesion
